# Strong Type 1, but Impaired Type 2, Immune Responses Contribute to *Orientia tsutsugamushi*-Induced Pathology in Mice

**DOI:** 10.1371/journal.pntd.0003191

**Published:** 2014-09-25

**Authors:** Lynn Soong, Hui Wang, Thomas R. Shelite, Yuejin Liang, Nicole L. Mendell, Jiaren Sun, Bin Gong, Gustavo A. Valbuena, Donald H. Bouyer, David H. Walker

**Affiliations:** 1 Department of Microbiology and Immunology, University of Texas Medical Branch, Galveston, Texas, United States of America; 2 Department of Pathology, Center for Biodefense and Emerging Infectious Diseases, Center for Tropical Diseases, Sealy Center for Vaccine Development, Institute of Human Infections and Immunity, University of Texas Medical Branch, Galveston, Texas, United States of America; University of Liverpool, United Kingdom

## Abstract

Scrub typhus is a neglected, but important, tropical disease, which puts one-third of the world's population at risk. The disease is caused by *Orientia tsutsugamushi*, an obligately intracellular Gram-negative bacterium. Dysregulation in immune responses is known to contribute to disease pathogenesis; however, the nature and molecular basis of immune alterations are poorly defined. This study made use of a newly developed murine model of severe scrub typhus and focused on innate regulators and vascular growth factors in *O. tsutsugamushi*-infected liver, lungs and spleen. We found no activation or even reduction in base-line expression for multiple molecules (IL-7, IL-4, IL-13, GATA3, ROR-γt, and CXCL12) at 2, 6 and 10 days post-infection. This selective impairment in type 2-related immune responses correlated with a significant activation of the genes for IL-1β, IL-6, IL-10, TNF-α, IFN-γ, as well as CXCR3- and CXCR1-related chemokines in inflamed tissues. The elevated angiopoietin (Ang)-2 expression and Ang-2/Ang-1 ratios suggested excessive inflammation and the loss of endothelial integrity. These alterations, together with extensive recruitment of myeloperoxidase (MPO)-expressing neutrophils and the influx of CD3^+^ T cells, contributed to acute tissue damage and animal death. This is the first report of selective alterations in a panel of immune regulators during early *O. tsutsugamushi* infection in intravenously inoculated C57BL/6 mice. Our findings shed new light on the pathogenic mechanisms associated with severe scrub typhus and suggest potential targets for therapeutic investigation.

## Author Summary

Scrub typhus is a life-threatening disease, often displayed as severe acute febrile illness. It is caused by infection with *O. tsutsugamushi*, a Gram-negative intracellular bacterium. Every year, approximately one million people are infected globally, especially in the Asia-Pacific region. Mechanistic studies of host immune responses are limited, partially due to the limited availability of suitable animal models and research facilities. We demonstrated here selective suppression in innate immune regulators, but activation of inflammatory cytokines, during the first few days of *O. tsutsugamushi* infection in mice. Importantly, these immune alterations correlated with acute tissue damage, disease progression, and animal death. As the first report describing dysregulation in innate immune responses in a murine model of severe scrub typhus, this study improves our understanding of disease mechanisms and opens new avenues for the exploration of treatment options.

## Introduction

Scrub typhus is an acute, febrile and often fatal disease, caused by infection with *O. tsutsugamushi* (formerly known as *Rickettsia orientalis* or *R. tsutsugamushi*) [1]. Every year, approximately one million people are infected globally, especially in the Asia-Pacific region. Humans are infected through the bites of the larva of trombiculid mites, and the Karp- and Gilliam-like genotypes account for 50% and 25% of all human infections, respectively [2], [3]. Scrub typhus is responsible for a large proportion of severe undifferentiated fevers, as well as up to 23% of all febrile episodes in rural endemic areas, and has relatively high mortality rates [4]. Some individuals can progress to persistent infection even after antibiotic treatment [5]. There are no effective vaccines for scrub typhus [3], [6]. Adaptive immunity following *O. tsutsugamushi* infection in humans appears short-lived [4], [7], [8], but the underlying mechanisms of protective immunity and its disappearance are unclear.

*O. tsutsugamushi* predominantly replicates in disseminated endothelial cells (EC), and degree in macrophages and dendritic cells in the inoculation eschar [9], [10], [11]. The bacteria invade cells by induced phagocytosis, escape from the phagosome, and replicate in the cytoplasm. The histopathological features include perivascular infiltration of monocytes/macrophages and T cells and generalized vasculitis involving tissues from several organ systems [12], [13]. Unlike other Gram-negative bacteria, *O. tsutsugamushi* lacks lipopolysaccharides (LPS) and peptidoglycan in its cell wall. Yet, both live and heat-killed *O. tsutsugamushi* are highly competent in stimulating proinflammatory cytokines [14], [15], presumably by stimulating the cytosolic NOD-like receptor family proteins and NF-κB-mediated pathways. Endothelial ICAM-1 expression and Th1 cell activation are often seen in patients [16], [17]. Pro-inflammatory responses are often crucial in bacterial clearance; however, excessive inflammation, apoptosis and TNF-α, IL-12, IL-10, CCL2 and CCL3 production can also contribute to acute respiratory distress syndrome, hepatitis and meningoencephalitis [18], [19]. Disease severity is often linked to high levels of these cytokines [20], [21], [22]. While these findings are important, the intra-peritoneal infection route and lack of information on early host responses to the pathogen in experimental animals have several intrinsic limitations in understanding scrub typhus pathogenesis [4], [19]. For instance, it is now understood that neutrophils and innate lymphoid cells (ILCs) in affected tissues are important gate keepers to pathogen infection and play a central role in modulating adaptive immune responses and tissue remodeling [23], [24], [25]. Based on their differentiation, transcription factors, and cytokine profiles, ILCs are divided into three functional groups [23], [25]. Reminiscent of Th1 cells, group 1 ILCs (ILC1) are dependent on T-bet and IL-15 and produce IFN-γ. ILC2 can produce Th2-associated cytokines such as IL-5 and IL-13 under the control of the transcription factors GATA3 and RORα. ILC3s are capable of producing Th17-associated cytokines under the control of ROR-γt. At present, there are no detailed studies of early events or alterations during *O. tsutsugamushi* infection.

We hypothesize that excessive Th1-type cytokines and inflammation in severe scrub typhus is due to immune dysregulation at early stages of the infection with *O. tsutsugamushi*. We have recently developed an infection model with endothelial tropism. The i.v. inoculation with *O. tsutsugamushi* Karp strain results in disseminated endothelial infection by *Orientia*, vasculitis, hemorrhage, interstitial pneumonia, and meningioencephalitis which closely resemble the pathological changes seen in scrub typhus patients [26]. Using this new model, we have investigated, in detail, aberrant cytokine responses in a lethal scrub typhus model that parallels what has been described in human scrub typhus [20], [21], [22]. This study opens new avenues for further examination of immune alterations and mechanisms.

## Materials and Methods

### Mouse infection and ethics statement

Female wild-type C57BL/6J mice were purchased from Jackson Laboratory and used in this study. Mice were maintained under specific pathogen-free conditions and used at 6–9 weeks of age following protocols approved by the Institutional Animal Care and Use Committee (protocols # 9007082B and 1302003) at the University of Texas Medical Branch (UTMB) in Galveston, TX. All mouse infection studies were performed in the ABSL3 facility in the Galveston National Laboratory located at UTMB; all tissue processing and analysis procedures were performed in the BSL3 or BSL2 facilities. All procedures were approved by the Institutional Biosafety Committee, in accordance with Guidelines for Biosafety in Microbiological and Biomedical Laboratories. UTMB operates to comply with the USDA Animal Welfare Act (Public Law 89-544), the Health Research Extension Act of 1985 (Public Law 99-158), the Public Health Service Policy on Humane Care and Use of Laboratory Animals, and the NAS Guide for the Care and Use of Laboratory Animals (ISBN-13). UTMB is a registered Research Facility under the Animal Welfare Act, and has a current assurance on file with the Office of Laboratory Animal Welfare, in compliance with NIH Policy.

*Orientia tsutsugamushi* Karp strain was used herein, and all infection studies were performed with the same bacterial stock prepared from liver extracts pooled from several infected mice, and infectious organisms were then quantified via a focus forming assay [26]. C57BL/6J mice were inoculated intravenously (i.v.) with *O. tsutsugamushi* (4.5×10^6^ FFU in 200 µl). Control mice were similarly injected with PBS, or liver extracts prepared from uninfected mice. Since no major differences were observed in mice receiving PBS or control liver extracts, data from these two types of controls were pooled in some experiments, and they were collectively referred to as controls in this report. At 0, 2, 6, and 10 days post-infection (dpi), serum and tissue samples were collected and inactivated for immediate or subsequent analyses.

### Quantitative reverse transcriptase PCR (qRT-PCR) analysis

Mouse tissues were collected in an RNA*Later* solution (Ambion, Austin, TX) at 4°C overnight to inactivate infectious bacteria and stored at −80°C for subsequent analyses. Total RNA was extracted from tissues by using an RNeasy mini kit (Qiagen, Valencia, CA) and digested with RNase-free DNase (Qiagen). cDNA was synthesized with the iScript cDNA synthesis kit (Bio-Rad Laboratories, Hercules, CA). The abundance of target genes was measured by qRT-PCR by using a Bio-Rad CFX96 real-time PCR apparatus, and a SYBR Green Master mix (Bio-Rad) was used for all PCR reactions. PCR reactions were started at 95°C for 3 min, followed by 39 cycles of 95°C for 10 sec, and 60°C for 10 sec, and ended with an elongation step at 72°C for 10 sec. Dissociation melting curves were obtained after each reaction to confirm the purity of PCR products. Relative abundance of mRNA expression was calculated by using the 2^−ΔΔCT^ method. Glyceraldehyde-3-phosphate dehydrogenase (GAPDH) was used as the housekeeping gene for all analyses of liver and spleen tissues, and β-actin was used as the housekeeping gene for all analyses of lung tissues. Primer sequences are listed in **Table S1**.

### Bacterial load determination

Bacterial loads were assessed by quantitative real-time PCR [27]. DNA was extracted using a DNeasy Kit (Qiagen, Gaithersburg, MD) from the tissue samples, and the bacterial load at each time point and for each organ sampled was determined by quantitative real-time PCR [27]. The gene for a 47-kDa protein was amplified by using specific primers [OtsuF630 (5′-AACTGATTTTATTCAAACTAATGCTGCT-3′) and OtsuR747 (5′-TATGCCTGAGTAAGATACGTGAATGGAATT-3′) (IDT, Coralville, IA)]. PCR products were detected with a specific probe [OtsuPr665 (5′-6FAM-TGGGTAGCTTTGGTGGACCGATGTTTAATCT-TAMRA) (Applied Biosystems, Foster City, CA)]. Bacterial loads were normalized to total nanogram (ng) of DNA per µL for the same sample, and data are expressed as the gene copy number of 47-kDa protein per picogram (pg) of DNA. The copy number for the 47-kDa gene was determined by known concentrations of a control plasmid containing single-copy insert of the gene. Gene copy numbers were determined via serial dilution (10-fold) of the control plasmid.

### Serum cytokine measurement

Serum samples were collected, inactivated via adding 0.09% sodium azide, and assayed for 32 proteins via Ray Biotech Mouse Cytokine Array C2 series, according to the manufacturer's instructions (AAM-CYT-2–8, Ray Biotech, Inc., Norcross, GA) or for IFN-γ by using an ELISA (eBioscience, San Diego, CA). Briefly, serum samples were diluted (1∶5) with a kit-provided buffer, and equal volumes were pooled from individual mice in each group (400 µl per sample). Membranes were blocked with blocking buffer, incubated with pooled sera (in 2 ml total volume) at 4°C overnight, washed, and then incubated with a cocktail of biotinylated antibodies at 4°C overnight. Membranes were washed and incubated with HRP-conjugated streptavidin for 2 h at room temperature. Bound antibodies were visualized by using enhanced chemiluminescence reagents (Ray Biotech). Signal intensity of each spot was scanned by ImageQuant and quantified with ImageQuant TL software (GE Healthcare, Pittsburgh, PA). Built-in positive spot controls were used to normalize signal intensities among different membranes prior to intra-membrane comparison.

### Oriential antigen preparation

*Orientia* were grown, as described previously [26]. Oriential antigen lysate was prepared from heavily (80–100%) infected Vero cell monolayers cultured in 150-cm^2^ flasks. Cell suspensions were collected in Oakridge high-speed centrifugation bottles was centrifuged at 22,000× g for 45 min at 4°C. The pellet was resuspended in sucrose-phosphate-glutamate (SPG) buffer (0.218 M sucrose, 3.8 mM KH_2_PO_4_, 7.2 mM KH_2_PO_4_, 4.9 mM monosodium L-glutamic acid, pH 7.0). To release the orientiae, host cells were lysed by sonication on ice by four 15-second pulses at 60% amplitude. Host cell debris was removed by centrifugation at 1000× g for 5 min. The supernatant was collected and centrifuged at 22,000× g for 45 min to pellet cell-free bacteria. The pellet was resuspended in PBS with 0.05% sodium azide and sonicated on ice by eight 15-second pulses at 60% amplitude to lyse the bacteria. After sonication, the remaining cell debris was removed by centrifugation at 1000× g for 5 min. The supernatant was harvested, passed through a 0.45-µm syringe filter, and protein concentration measured.

### Antibody isotype determination

To determine the antibody isotype profile, 96-well Maxisorp plates (Nunc, Rochester, NY) were coated with oriential antigen (50 µg/ml in PBS) at 4°C overnight. After blocking, plates were incubated with individual serum samples (1∶100 dilution) for 1 h at room temperature, and then with alkaline phosphatase-conjugated goat anti-mouse IgM, IgG1, or IgG2c (1∶300, SouthernBiotech, Birmingham, AL). Color was developed with the phosphatase substrate solutions (KPL, Gaithersburg, MD). OD values at 650 nm were measured with a VersaMax microplate reader (Sunnydale, CA).

### Immunohistochemistry (IHC)

All tissues were fixed in 10% neutral-buffered formalin and embedded in paraffin, and sections (5-µm thickness) were stained with hematoxylin and eosin or processed for antibody (Ab) staining. For IHC staining, sections were deparaffinized and treated with an antigen-retrieval solution (Dako, Carpinteria, CA) at 98°C for 25 min, a peroxidase-blocking solution (Dako) for 15 min, and then with a streptavidin-biotin blocking solution (Vector Laboratories, Burlingame, CA) for 1 h. Sections were incubated for 2 h at room temperature with one of the following antibodies: rabbit anti-*O. tsutsugamushi* Karp strain polyclonal antibody (pAb, 1∶500); rat anti-mouse neutrophil monoclonal antibody (mAb, 1∶25, clone 7/4, Caltag Laboratories, Buckingham, UK); rabbit anti- myeloperoxidase (MPO) pAb (1∶25, Abcam); or rabbit anti-mouse CD3 mAb (1∶25, Abcam, Cambridge, MA). Biotinylated goat anti-rat or anti-rabbit secondary antibodies (1∶200, Vector) were incubated on the sections for 30 min. Sections were stained with alkaline phosphatase-conjugated streptavidin (1∶200, Vector), Vector Red alkaline phosphatase substrate, and counterstained with hematoxylin (Sigma, St. Louis, MO). Reagent negative controls consisted of samples in which primary Ab was replaced with normal rat or rabbit IgG. Sections were dehydrated, mounted in Permount (Vector), and imaged under an Olympus BX53 microscope.

### Statistical analysis

Data were presented as mean ± standard errors of the mean (SEM). Differences between individual treatment and control groups were determined by using Student's t test. One-way ANOVA was used for multiple group comparisons. Statistically significant values are referred to as *, *p*<0.05; **, *p*<0.01.

## Results

### Imbalance of type 1 and type 2 immune responses in *O. tsutsugamushi*-infected mice

**Figure 1:**
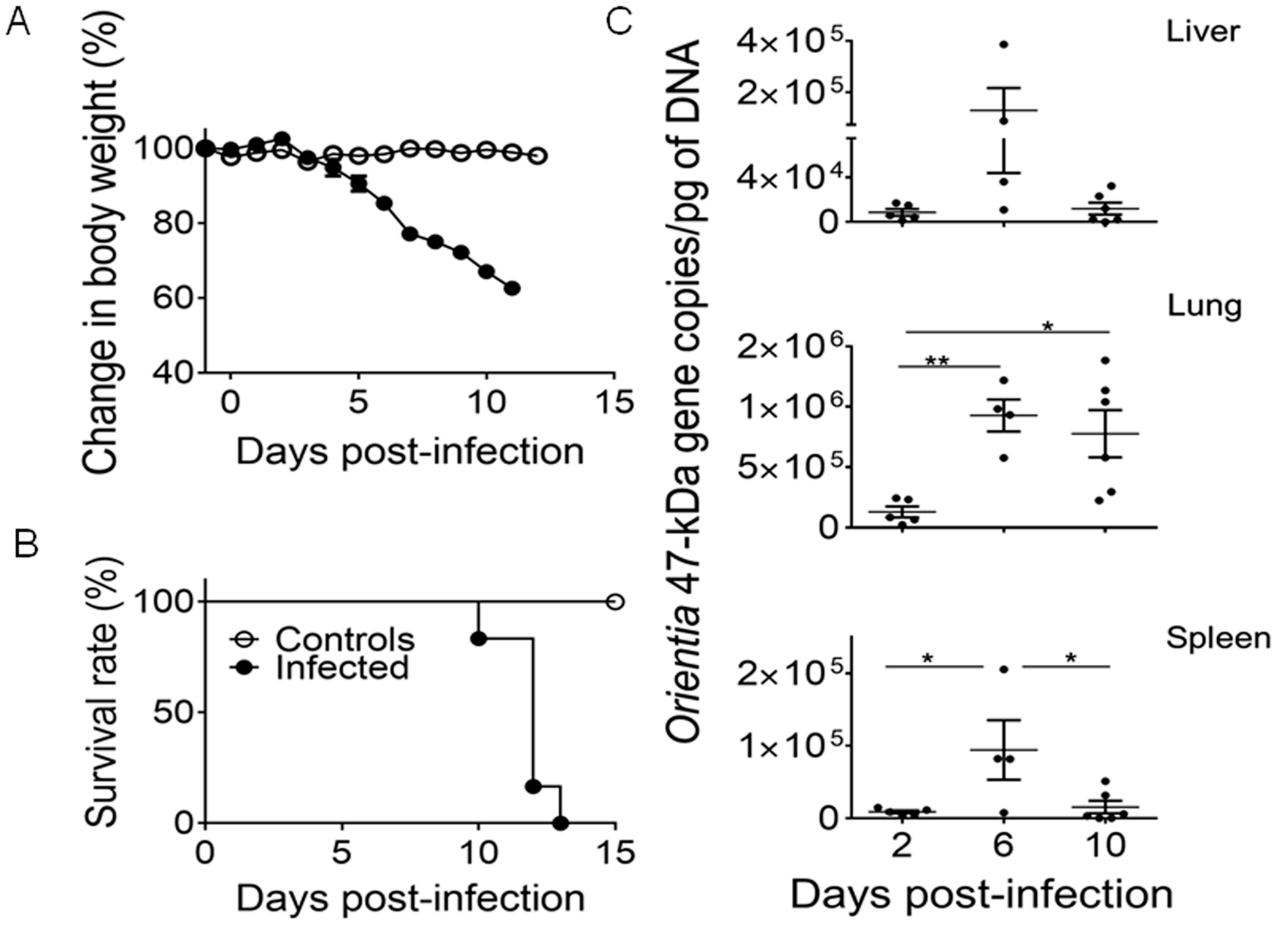
Body weight change, survival, and bacterial load kinetics of mice inoculated with *O. tsutsugamushi* intravenously. Female C57BL/6J mice (5/group) were inoculated i.v. with *O. tsutsugamushi* Karp stain (4.5×10^6^ FFU, solid circles) or PBS (open circles). Animals were monitored for signs of illness and weighed daily throughout the course of infection. Infected mice began losing weight 4 dpi with signs of illness (hunched posture, ruffled fur) starting at 6 dpi (**A**). Weight loss continued throughout infection with the mice succumbing to disease 10–13 dpi (**B**). Corresponding with the weight loss and development of signs of illness, mice sacrificed at 6 dpi had the highest bacterial loads in the liver, lungs, and spleen (**C**). *, *p*<0.05; **, *p*<0.01.

The newly developed mouse model of lethal *O. tsutsugamushi* infection prompted us to investigate host immune responses. Following inoculation of *O. tsutsugamushi* Karp strain (4.5×10^6^ FFU), mice maintained a normal body weight during the first 4 days post-infection (dpi), but began to lose 15–25% of body weight between 5 and 6 dpi (Fig. 1A). Infected mice lost approximately 34–38% of body weight by 10 dpi, and all mice had expired by 12–13 dpi (Fig. 1B). Bacterial loads in the liver, lungs and spleen peaked at 6 dpi, which coincided with disease onset, i.e., hunched posture, ruffled fur, and initial weight loss (Fig. 1C). To examine the underlying pathophysiological mechanisms, we focused our studies on the incubation period (2 dpi), the disease onset (6 dpi), and the terminal stage (10 dpi) prior to animal death. All data were compared with age-matched mock controls (see Materials and Methods section).

**Figure 2:**
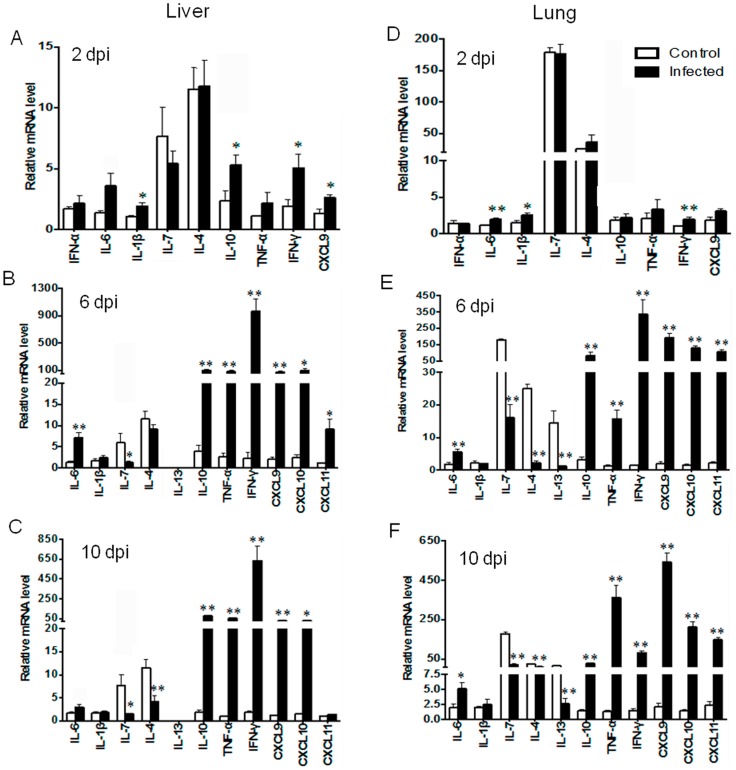
Liver and lung gene expression following infection. C57BL/6J mice (4–5/group) were inoculated i.v. with *O. tsutsugamushi* Karp stain (4.5×10^6^ FFU, black bars) or with PBS (open bars). At 0, 2, 6 and 10 dpi, total RNA was extracted for qRT-PCR analyses of indicated genes. (**A–F**) Data are shown as mean ± SEM in each group and presented as “relative mRNA levels” (after normalization to the house-keeping genes). Representative results are shown from two independent studies with similar trends. *, *p*<0.05; **, *p*<0.01.

The gene expression profiles in the liver and lung tissues are shown in Fig. 2. A significant induction of IL-1β, IL-6, IL-10, IFN-γ, and CXCL9 in these organs at 2 dpi suggested innate immune sources for these cytokines. No increase in IFN-α was observed in infected liver and lung tissues at 2 dpi, suggesting the dispensable role of IFN-α at this time. At 6 dpi, we found three distinct, but highly consistent, trends in infected liver and lung tissues as compared to mock tissues. Firstly, there was a remarkable increase in the levels of type 1 cytokines, including TNF-α (15-fold), IFN-γ (300-fold), and CXCL9-11 (∼100-fold). Secondly, there was no f IL-4 elevation in the liver, but a significant reduction in the baseline expression levels of IL-7, IL-4, and IL-13 in infected lung tissues. Thirdly, IL-10 expression was significantly increased in infected liver and lung tissues, and its trends of expression were consistent with those of TNF-α, IFN-γ, and CXCL9-11. Importantly, the elevation of IL-10, TNF-α, IFN-γ, CXCL9 and CXCL10 was consistent and maintained at 10 dpi, while the reduction of IL-7, IL-4, and IL-13 was sustained. The IL-1β levels were comparable between uninfected and infected groups at 6 and 10 dpi, implying a limited role for this cytokine in the adaptive immune responses.

**Figure 3:**
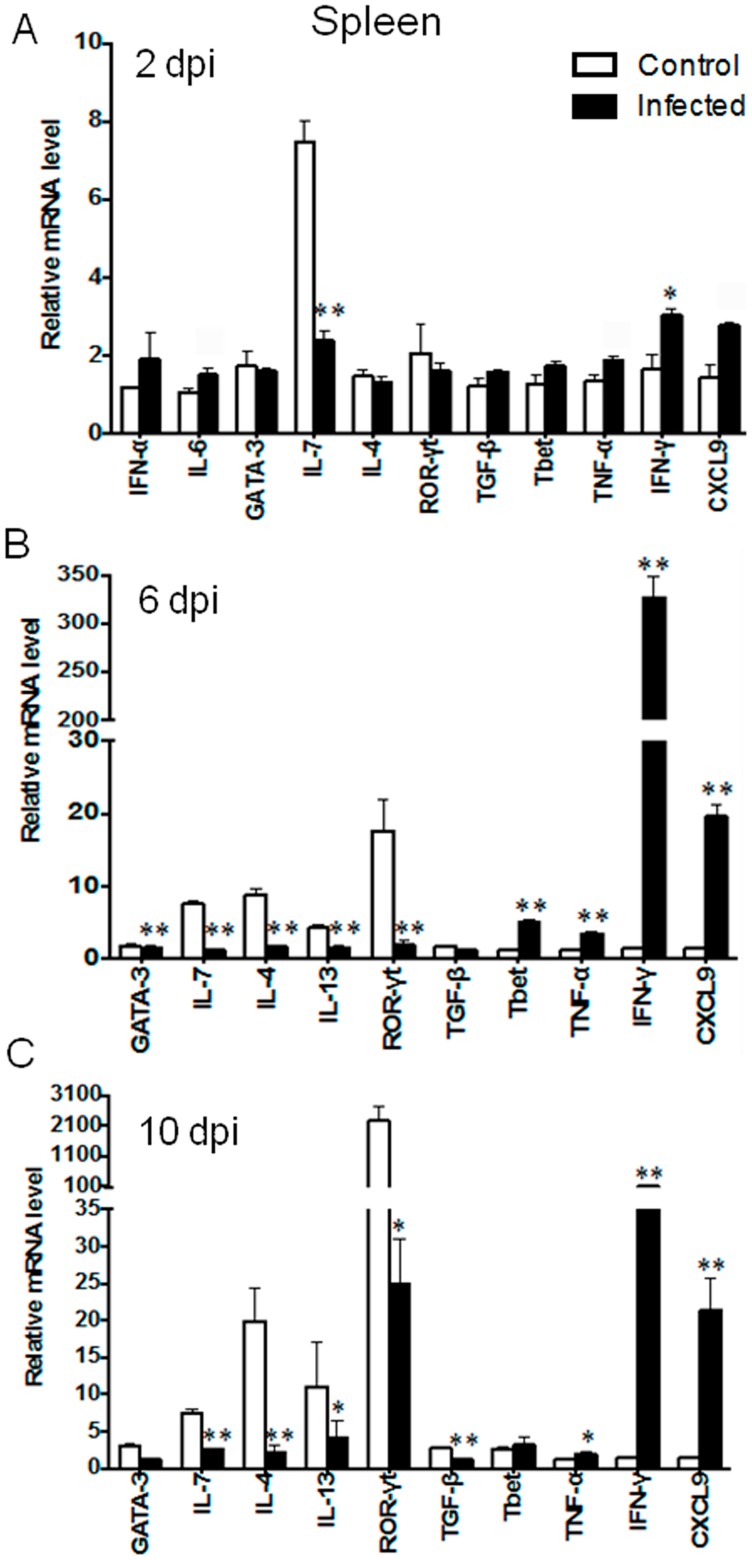
Splenic gene expression following infection. Mice (4–5/group) were inoculated with *O. tsutsugamushi* Karp stain, and spleen tissues collected at days 2 (**A**), 6 (**B**) and 10 (**C**) of infection and analyzed, as described in ***Fig. 1***. Data are shown as mean ± SEM in each group and are presented as “relative mRNA levels” (after normalization to the housekeeping genes). Data are presented as fold changes (log_2_) in comparison to the levels in their corresponding control samples (0 dpi). Representative results are shown from two independent studies with similar trends. *, *p*<0.05; **, *p*<0.01.

To expand these findings, we examined splenic levels of signature type 1 cytokines (TNF-α, IFN-γ, and CXCL9), type 2 cytokines (IL-7, IL-4, and IL-13), as well as their corresponding transcription factors (T-bet, GATA3, and ROR-γt) that are critical for the development and functions of ILC and Th subsets [23], [28]. The significant elevation of IFN-γ, but a reduction of IL-7, at 2 dpi suggested dysregulated ILC subsets at early stages of infection (Fig. 3A). At 6 dpi, we found a modest, but significant, elevation of T-bet and TNF-α, as well as a marked increase in IFN-γ (300-fold) and CXCL9 (15-fold). As expected, the expression levels for IL-7, IL-4, IL-13, and GATA3 were significantly lower than those in the controls at 6 dpi (Fig. 3B). There was an approximately 8-fold reduction in ROR-γt transcripts at both 6 and 10 dpi. The strong type 1, but impaired type 2 responses, continued through 10 dpi (Fig. 3C). Such alterations were consistent with marked induction of IgG2c in the sera of infected mice (Fig. S2). Therefore, we conclude that there is an altered immune homeostasis with selective impairments in type 2 cytokines and transcriptional factors during *O. tsutsugamushi* infection.

### Elevated type-1 cytokines in the sera of infected mice

To further investigate the above findings, we collected serum samples at 0, 2, 6 and 10 dpi and measured 32 molecules by using mouse cytokine arrays (see Materials and Methods). *O. tsutsugamushi* infection induced no or marginal increase in IL-4, IL-5, IL-13, IL-10, IL-17, and TNF-α levels in sera (Fig. S1A). However, we found 2- to 5-fold increases in IL-6, IFN-γ, G-CSF, TIMP1, CCL5, CCL12, and IL-12p70 levels at 6 dpi (Fig. S1, B and C). ELISA studies confirmed high IFN-γ levels in the sera at 6 dpi, which markedly decreased at 10 dpi (Fig. S1D). These data were consistent with our RT-PCR results, indicating strong type 1-dependent immune responses, but weak type 2- and IL-17 immune responses, in the infected mice. To further assess the type 1-dependent immune response, we evaluated the *in vivo* production of type 1 (IgG2c) and type 2 (IgG1) antibodies, as well as IgM, during lethal *Orientia* infection. Significant levels of IgM were detected via ELISA at 10 dpi compared to uninfected mice (Fig. S2A). IgG2c levels were significantly higher than IgG1 levels in lethally infected animals (Fig. S2B).

### Neutrophil and T cell recruitment and activation in infected tissues

**Figure 4:**
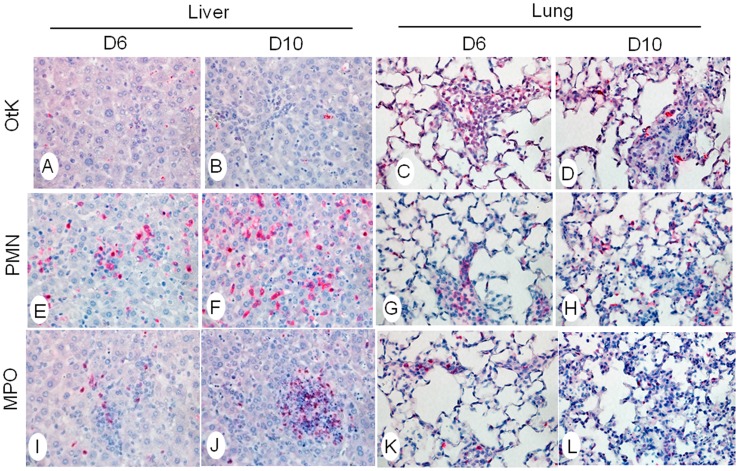
Neutrophil recruitment and activation in the liver and lungs. Mice (4–5/group) were inoculated with *O. tsutsugamushi* Karp stain (OtK) (**A–D**), as described in ***Fig. 1***. Tissue sections were collected at indicated times, processed, and stained for bacteria, neutrophils (PMN) (**E–H**), or myeloperoxidase (MPO) (**I–L**). Positive staining is in red. Images were photographed at 40X.

To better understand the impact of altered cytokine/chemokine responses during the infection, we examined cellular responses in the tissues, and we initially focused on neutrophils. Spleen sections of uninfected control (D0) mice contained no bacterial staining (Fig. S3A), and some neutrophils were sparsely distributed in red pulp between the germinal centers (Fig. S3, D), together with relatively low levels of MPO (Fig. S3, G), a lysosomal protein stored in azurophilic granules of neutrophils. At 6 and 10 dpi, *O. tsutsugamushi* staining was evident (Figs. S3, B and C). Neutrophils increased in numbers, and the clusters of MPO-positive neutrophils increased in size and staining intensity, especially at 10 dpi, accompanied by disorganization of germinal centers (Fig. S3, E, F, H, I). In the liver, bacterial staining was readily detected at 6 and 10 dpi (Fig. 4, A and B). Infiltrating neutrophils formed clusters near blood vessels at 6 dpi, and neutrophils were detected throughout the liver parenchyma at 10 dpi (Figs. 4E and F). In some areas, the neutrophil clusters became more concentrated, and the quantity of MPO-positive neutrophils increased (Fig. 4I and J). In the lungs, oriential antigen staining in endothelial cells was relatively more abundant than that seen in the spleen and liver, which correlated with bacterial burden data (Fig. 1C). Loss of alveolar air space and presence of interstitial pneumonia were evident at 6 dpi and more severe at 10 dpi (Fig. 4, right columns). These pathological and immune changes were consistent with type 1-skewed immune responses in *O. tsutsugamushi*-infected mice (Figs. 2–4).

**Figure 5:**
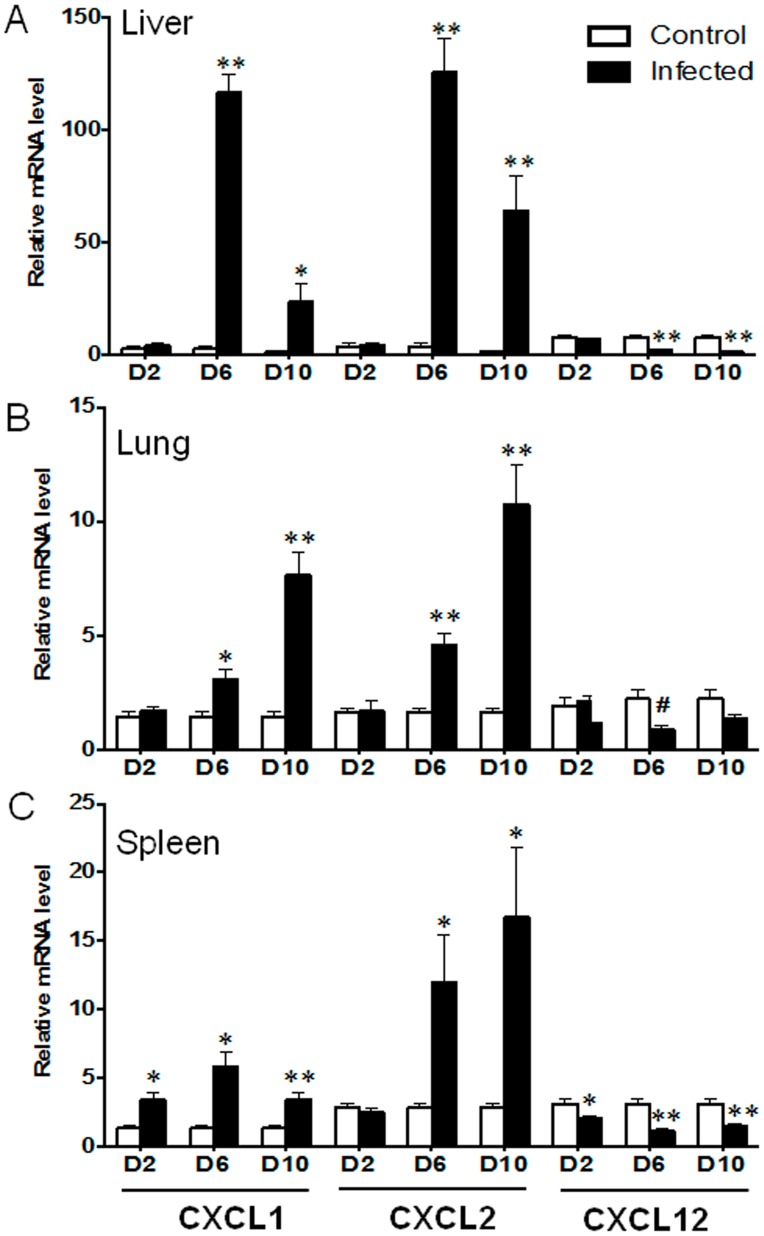
Neutrophil-recruiting chemokine expression during the infection. Mice (4–5/group) were inoculated with *O. tsutsugamushi* Karp stain, as described in ***Fig. 1***. Samples were collected from the liver (**A**), lungs (**B**) and spleen (**C**) at 0, 2, 6, and 10 dpi and analyzed via qRT-PCR for gene expression. Data are shown as the mean ± SEM in each group and presented as “relative mRNA levels” (after normalization to the house-keeping genes). *, *p*<0.05; **, *p*<0.01.

To further examine the effect of immune mechanisms on neutrophil recruitment and activation, we analyzed the expression levels of CXCL1, CXCL2 and CXCL12. CXCL1 expression levels were significantly increased in the spleen at 2 dpi, but not in the liver and lungs (Fig. 5). At 6 and 10 dpi, CXCL1 and CXCL2 levels were significantly elevated in the liver, lungs and spleen, which correlated with the extensive influx and activation of neutrophils in the infected tissues (Figs. 4 and S2). However, CXCL12 expression was markedly suppressed in the spleen at 2 dpi, which were similar to the patterns in the liver and lungs at 6 and 10 dpi. Since the CXCL12/CXCR4 axis is involved in the maintenance of the Th2 cell function [29], [30], this CXCL12 suppression was consistent with impaired Th2 responses in infected mice (Figs. 2 and 3).

**Figure 6:**
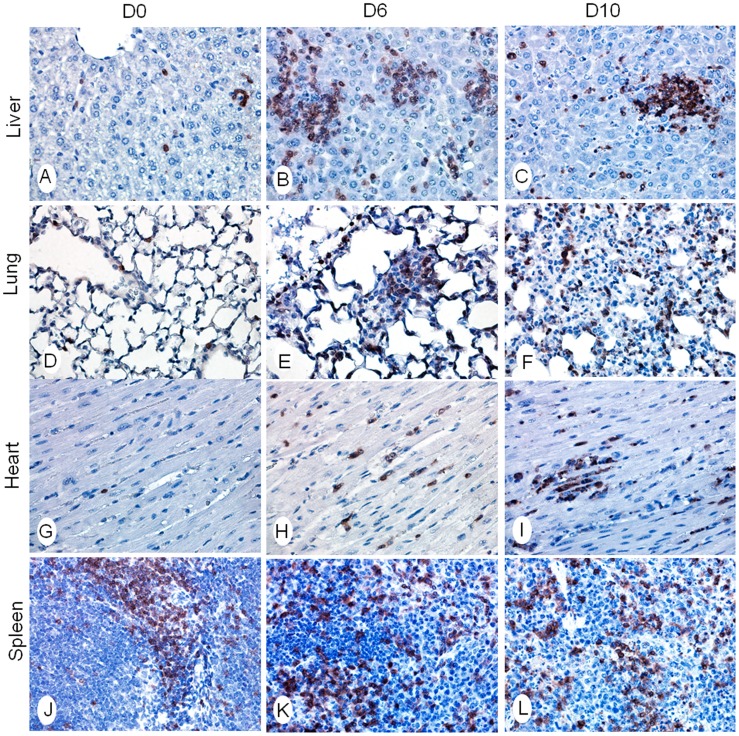
CD3^+^ T cell distribution in infected tissues. Mice were inoculated with *O. tsutsugamushi* Karp stain, as described in ***Fig. 1***. Indicated tissue sections were collected at 0, 6 and 10 dpi and stained for anti-CD3 (brown) (Liver: **A–C**; Lung: **D–F**; Heart: **G–I**; Spleen: **J–L**). Images were photographed at 40X.

Since neutrophils are the first line of host defense and an important regulator for adaptive immune responses, we then examined the recruitment and distribution of T cells. Control mice had minimal numbers of CD3^+^ T cells in the liver, lungs, and heart, and a normal distribution of T cells in the spleen (Fig. 6 and Fig. S4). CD3^+^ T cell clusters of variable sizes were detected in these tissues at 6 dpi. T cell infiltration appeared to be intensified and widely distributed in the liver, lungs, and heart at 10 dpi, which correlated with the continued loss of alveolar air space in the lungs and accumulation of damaged cells in these organs. In addition, we found a marked rearrangement of T- and B-cell zones in the spleen (Fig. S4 j–l), which was suggestive of extensive lymphocyte activation during the infection.

### Endothelial cell (EC) activation and dysregulation during the infection

**Figure 7:**
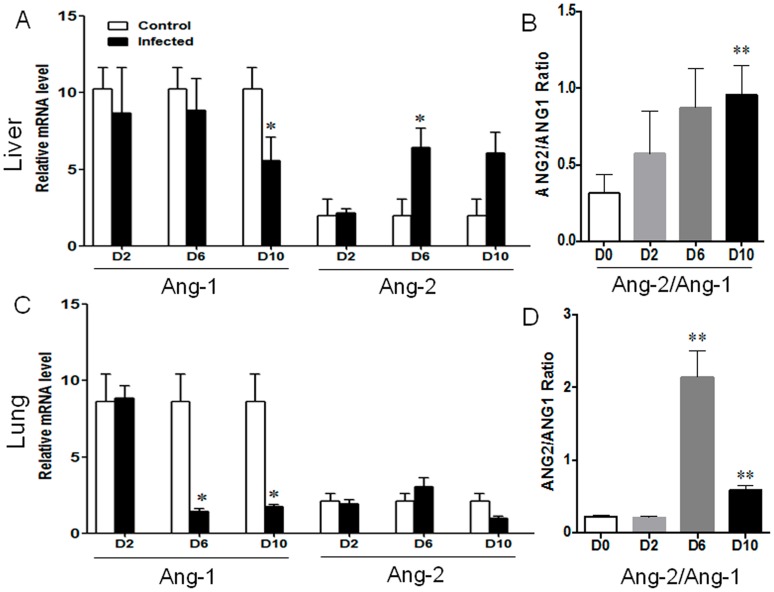
Endothelial cell activation and stress in the liver and lungs. Mice (4–5/group) were inoculated with *O. tsutsugamushi* Karp stain, as described in ***Fig. 1***. Liver and lung tissues were collected at 0, 2, 6, and 10 dpi and analyzed via qRT-PCR for gene expression. (**A, C**) Data are shown as mean ± SEM in each group and are presented as “relative mRNA level” (after normalization to the housekeeping genes). (**B, D**) The Ang-2/Ang-1 ratios of individual samples were calculated based on the corresponding data in **A** and **C**. *, *p*<0.05; **, *p*<0.01.

Once disseminated, *O. tsutsugamushi* preferentially replicates in EC, causing vasculitis and multiple organ dysfunction [10], [31]. Based on our cytokine and IHC results, we speculated that mortality resulted from possible compromised endothelial cell function. Accordingly, we examined angiopoietin (Ang) 1 and Ang 2. Ang 1 is constitutively expressed, and regulates and promotes endothelial cell quiescence. Ang-2, on the other hand, is a regulator stored in endothelial cells and leads to endothelial activation upon release by infection or inflammatory stimuli [32], [33]. As expected, normal livers and lungs maintained steady-state levels of Ang-1, but relatively low Ang-2 levels (Fig. 7). During *O. tsutsugamushi* infection, however, Ang-1 levels were markedly decreased in the liver and lungs, while Ang-2 levels were markedly increased in the liver. Compared to mock controls, the Ang-2/Ang-1 ratios were significantly increased in the liver and lungs at 6 and 10 dpi (Figs. 7B, D). Collectively, these data suggest EC activation and dysregulation in the infected mice.

## Discussion

By using a newly developed murine model of severe scrub typhus, this study demonstrated dysregulated innate and adaptive immune responses and their association with pathogenesis and lethality. Our data indicate that i.v. inoculation of a lethal dose of *O. tsutsugamushi* resulted in aberrant type 1- and type 2-related immune responses, as well as altered levels of CXCL12, Ang-1 and Ang-2. Overzealous type 1, but impaired type 2, innate and adaptive responses were linked to endothelial cell dysfunction and acute tissue damage, leading to mortality. Infiltration of innate cell neutrophils and adaptive T cells in various tissues of different infection stages were observed. This study presents immunological correlates of severe scrub typhus and further validates the intravenous inoculation as a valuable model to study scrub typhus [26].

The current understanding of anti-*Orientia* immune responses in vivo has been mostly derived from mouse studies via intraperitoneal inoculation of the bacteria, some of which measured cytokine responses in peritoneal lavages of infected mice [34]. These studies demonstrated significantly higher levels of pro-inflammatory cytokines in infected mice; however, it is difficult to evaluate early events and tissue-specific responses in those studies. Here, an intravenous inoculation model (Fig. 1), which results in a hematogensously disseminated endothelial infection paralleling human disease [26], was used. This model resembles early events after *Orientia* escapes the site of mite attachment and primarily infects endothelial cells, as well as the severity of disease. This i.v. route of challenge has been used in development of murine models for spotted fever group ricketssioses with instructive results with immunology and pathology paralleling human diseases [35], [36], [37], [38]. To our knowledge, there are no detailed reports that examine the immunological response to *Orientia* infection at the tissue level in pathologically relevant organs.

Currently, there is little evidence as to whether *Orientia* infection or replication directly or selectively suppresses type 2 responses. It is known that cytokines produced by Th1 cells can inhibit differentiation of naïve CD4^+^ T cells into Th2 cells, and *vice versa* [39]. Reported in vitro studies indicated that *R. conorii*-infected, bone marrow-derived dendritic cells can up-regulate IFN-γ, but down-regulate IL-4, production by CD4^+^ T cells [40], implying a cross-regulation of types 1 and 2 responses in *R. conorii* infection. We report here that type 1 responses peaked at or around 6 dpi and remained elevated throughout the course of the disease. It was surprising that type 2 cytokines remained suppressed in all examined tissues at 10 dpi, even when bacterial loads in the liver and spleen had decreased at 10 dpi, and when type 1 responses were somewhat restrained. Regardless of whether suppressed type 2 responses were mediated by the host, the pathogen, or both, we believe that this dysregulation contributed to the pathology, severity, and host death.

The pro-inflammatory response is crucial to the control of pathogens, but if it is not finely controlled dire consequences can occur. Cytokine storm is characterized by abundant release of pro-inflammatory cytokines, such as IFN-γ, TNF-α and IL-6, as well as the anti-inflammatory cytokines such as IL-10; however, its mechanism of induction in oriential infection is not totally understood. We have found here that infection with *O. tsutsugamushi* evoked an early up-regulation of innate IFN-γ, which would promote parenchymal cells to express CXCL9 and CXCL10 chemokines. Similar trends have been described for *R. conorii* [41], [42]. Adaptive immune cells (including T cells) were attracted to the site of infected tissues, resulting in an uncontrolled type 1 immune reaction at 6 dpi. This cytokine storm caused the severe tissue injury and finally led to animal death at 10–13 dpi. Importantly, type 2- and IL-17-mediated immune reactions were largely silent or down-regulated during infection. Impaired expression of transcriptional factors GATA3 and ROR-γt resulted in failure to differentiate type 2 cells and/or IL-17-producing cells, leading to uncontrolled expression of TNF-α, IFN-γ and CXCR3 chemokines. Our finding of the fatal cytokine storm in *O. tsutsugamushi* infection was intriguing and suggested a therapeutic approach of rebalancing the type 1 and type 2 responses in scrub typhus.

IL-7 is a hematopoietic growth factor, critical for differentiation of pluripotent stem cells and important for the development and function of ILC2 and ILC3 cells [23], [25], [43]. The role of IL-7 in *O. tsutsugamushi* infection is enigmatic. Early and sustained impairment in IL-7 expression in the liver, lungs and spleen, followed by the widespread suppression of IL-7, IL-4, and IL-13, was observed in multiple organs (Figs. 2 and 3). It is possible that IL-7 impairment may lead to dysfunction of both ILC2 and ILC3 cells, which collectively lead to impaired IL-4, IL-5, IL-13, and IL-17 expression in *O. tsutsugamushi*-infected mice. The absence or marginal detection of IL-4, IL-5, IL-13, and IL-17 in sera at 2–10 dpi also supports the notion of suppressed Th2- and Th17-type responses (Fig. S1). Further investigation is needed to address the *in vivo* role of IL-7 in this model.

Of note, we found that IL-10 expression levels closely paralleled those of IFN-γ and TNF-α in the examined tissues and time points (Figs. 2 and 3). Given that IL-10 can be produced by different cells in the innate and adaptive immune arms, and that some Th1 and Treg cell subsets can simultaneously produce IFN-γ and IL-10 during infection (e.g., *Leishmania* and *Rickettsia*) [44], [45], [46], it will be interesting to examine the roles of IL-10 during *O. tsutsugamushi* infection.

Since the dysregulation of cytokines was detected in different tissues, we wanted to determine the infiltrative immune cell subpopulations during infection. As neutrophils are the first line of defense, we hypothesized that they play a critical role in this model and in scrub typhus pathology. We demonstrated a significant increase in neutrophil chemokines CXCL1/2, but not the Th2 chemokine CXCL12, as well as MPO^+^ neutrophils influx into infected tissues. Consistent with our findings, CXCL2 has also been shown to be expressed in the peritoneal lavages of lethal i.p. infected mice, as well as continual neutrophil detection in the peritoneal lavages [34]. In the present study, we did not further define the innate/early sources of IFN-γ, because ILC1, NK, NKT, γδ T cells, and neutrophils are known to produce IFN-γ [23], [25], [47], while CD3^+^ T cells are the main source of IFN-γ in the adaptive responses. Future studies, especially single cell-based analyses, are warranted to define the early source(s) of IFN-γ and the role of neutrophils in initiating the cytokine storm. Our IHC studies have shown extensive infiltration of CD3^+^ T cells in the liver, lungs, heart and spleen (Figs. 6 and S4). Activated CD3^+^ T cells can produce a high level of IFN-γ and other cytokines, which further enhance the production of CXCR3^+^ Th1 cells and CXCL9/10/11 chemokines, forming a positive feedback loop. Thus, modulating the level and timing of IFN-γ and CXCL9/10/11 production may be a potential therapeutic option in scrub typhus.

Finally, we showed that endothelial cell activation occurred at the early stage of *O. tsutsugamushi* infection. The CXCL12/CXCR4 axis is important for the maintenance of Th2 bias during physiological and pathological conditions [29], [30], [48], as well as for angiogenesis by recruiting endothelial progenitor cells from the bone marrow [49]. Our findings of elevated CXCL9-11, but impaired CXCL12 expression (Figs. 2, 3, and 5), imply excessive Th1 cell recruitment and impaired Th2 cell recruitment in inflamed tissues. It is also possible that CXCL12 suppression, in conjunction with altered Ang-2/Ang-1 ratios (Fig. 7), collectively contribute to endothelial dysfunction in our model. Angiopoietins are vascular growth factors for embryonic and postnatal angiogenesis, and impairment in Ang-1 and Ang-2 is known to be linked to the fatality rates of cerebral malaria and complicated *E. coli* O157:H7 infection in patients [32], [33]. Depressed levels of Ang-1 (endothelial integrity) and elevated Ang-2 expression and Ang-2/Ang-1 ratio were suggestive of endothelial cell stress and activation during *O. tsutsugamushi* infection. Otterdal, *et al.*, have recently shown that endothelial activation is strongly associated with the severity of scrub typhus [50]. Targeting this dysfunction in severe scrub typhus may be explored in future therapeutic approaches and studies.

Our most important finding is that *O. tsutsugamushi* infection resulted in broad and sustained down-regulation of IL-7, IL-4, IL-13, GATA3, ROR-γt and CXCL12. These impairments were accompanied by marked activation of IFN-γ, CXCR3 chemokines, and markers for endothelial activation at the onset of clinical disease. Our findings of profound immune imbalance in inflamed tissues, but modest changes in serum samples (Fig. S1), are noteworthy. The lack of evidence for suppressed type 2 responses in serum samples may not be surprising for several reasons. First, qRT-PCR detection of cytokine transcripts in inflamed tissues was more sensitive than a bioplex-based assay for proteins in sera. Secondly, serum measurements offer a snapshot of what is happening during infection, but only those cytokines that are being highly secreted at the time of collection will be detectable. Finally, target cells with high-affinity cytokine receptors may bind and consume secreted cytokines, reducing cytokine levels in serum. Additional studies with sublethal infection models and gene-targeted mice will help define whether deficient type-2 responses were due to bacterium-mediated alterations (insufficient stimulation or selective inhibition), or Th1-mediated down-regulation, or both. Nevertheless, this study validates a novel mouse model of scrub typhus, and, for the first time, reveals an immune imbalance during *O. tsutsugamushi* infection.

In summary, we have used an endothelial-target infection model of severe scrub typhus for a study of host immune responses triggered by *O. tsutsugamushi*. Our findings of strong activation in type-1 cytokines and antibodies, but silencing of type-2 immune responses and lack of type 2 antibodies, imply possible dysregulation of innate immune responses, which help explain acute tissue injury and animal death. Our findings offer new insights into the pathogenesis of scrub typhus. This study calls for a detailed investigation of host innate responses, and the findings also implicate potential therapeutic targets for severe scrub typhus.

## Supporting Information

Figure S1 **Serum cytokine levels during the infection.** Mice (4–5/group) were inoculated with bacteria, as described in ***Fig. 1***. Sera were collected at indicated time points, pooled for each group, and measured for protein levels by using mouse cytokine arrays (**A–C**). Relative spot intensities were normalized to in-kit negative and positive controls. Spot intensity data are presented in three arbitrary groups in **A** (<5×10^5^ intensity units), **B** (<20×10^5^ intensity units), and **C** (<80×10^5^ intensity units), respectively. (**D**) IFN-γ levels in pooled serum samples were measured by an ELISA. ND, not detectable.
(TIF)

Figure S2 **Antibody isotypes during** ***Orientia*** **infection.** Mice (4–5/group) were inoculated with bacteria, as described in ***Fig. 1***. Sera were collected at 10 dpi, and antibody isotypes were determined by ELISA (IgM (**A**); IgG1-vs-IgG2c (**B**)).
(TIF)

Figure S3 **Neutrophil expansion and activation in the spleen.** Mice (4–5/group) were inoculated with *O. tsutsugamushi* Karp stain (OtK) (**A–C**), as described in ***Fig.*** *1*. Spleen sections were collected at 0, 2, 6, and 10 dpi, processed, and stained by IHC for bacteria, neutrophils (PMN) (**D–F**), or myeloperoxidase (MPO) (**G–I**). Images were photographed at 40×. Positive staining is in red. Examples of bacterial staining are marked in circles.
(TIF)

Figure S4 **CD3^+^ T cell distribution in** ***O. tsutsugamushi*** **-infected tissues.** Mice (4–5/group) were inoculated with *O. tsutsugamushi*, as described in ***Fig. 1***. Tissue sections were collected from the liver (**a–c**), lungs (**d–f**), heart (**g–i**), and spleen (**j–l**) at 0, 6 and 10 days post-infection and stained for anti-CD3. Images were photographed at 10×. The most remarkable changes were: 1) the extensive distribution of CD3^+^ T cells in the liver at 10 dpi (in comparison to 6 dpi), 2) the marked loss of alveolar space in the lungs at 10 dpi (due to extensive inflammatory responses), and 3) the extensive reorganization of T- and B-cell zones in the spleen.
(TIF)

Table S1 **Real-time PCR primers of murine genes.** The primer sequences for mouse genes analyzed in this study are listed (5′ to 3′ direction).
(DOCX)
