# Supplementary figures and images for: Strong Type 1, but Impaired Type 2, Immune Responses Contribute to Orientia tsutsugamushi-Induced Pathology in Mice
Source: PLoS Negl Trop Dis. 2014 Sep 25;8(9):e3191. doi: 10.1371/journal.pntd.0003191 (PMC4177881; doi:10.1371/journal.pntd.0003191)

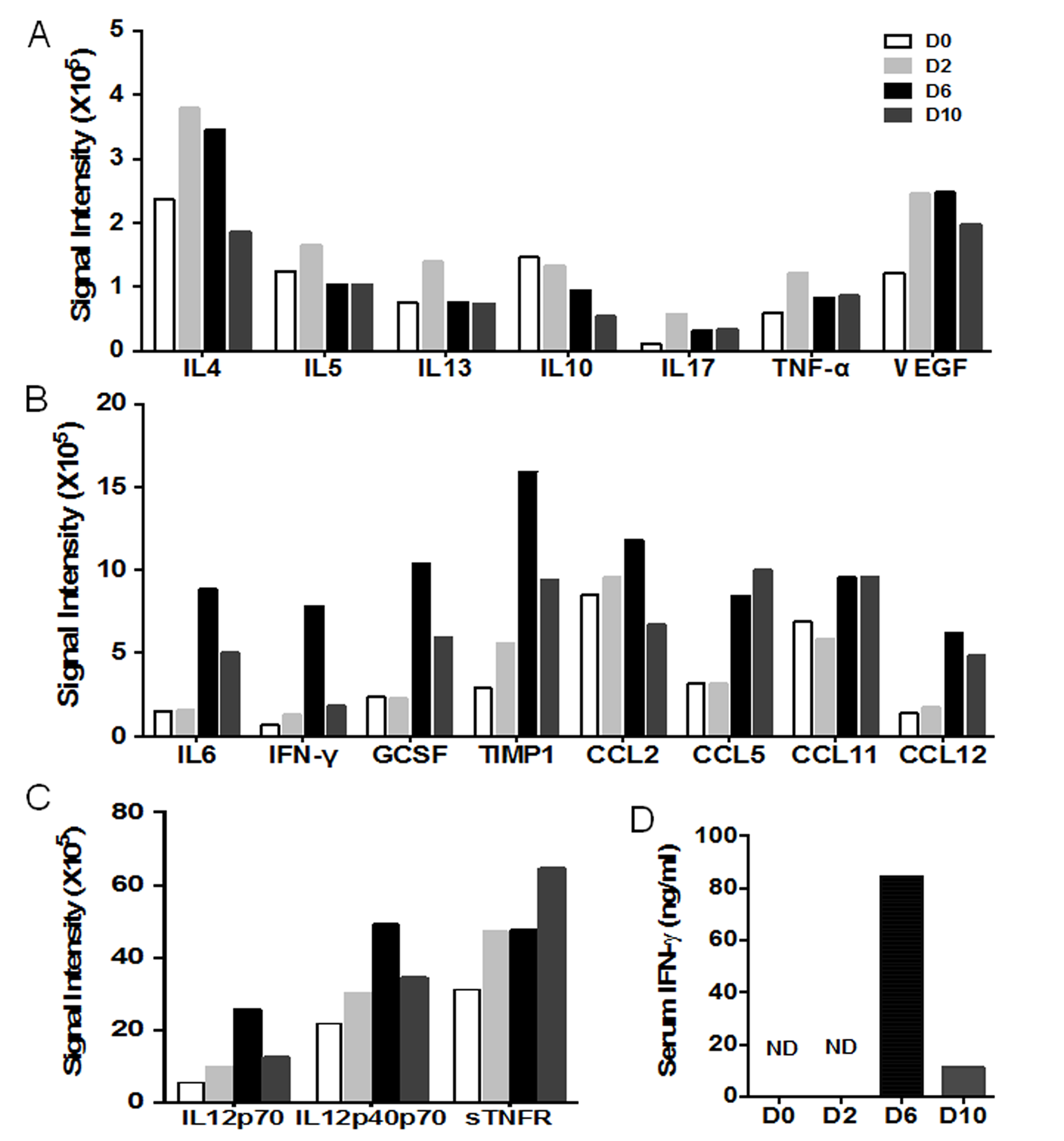

Supplement: Figure S1 — Serum cytokine levels during the infection. Mice (4–5/group) were inoculated with bacteria, as described in Fig. 1 . Sera were collected at indicated time points, pooled for each group, and measured for protein levels by using mouse cytokine arrays (A–C). Relative spot intensities were normalized to in-kit negative and positive controls. Spot intensity data are presented in three arbitrary groups in A (<5×10^5^ intensity units), B (<20×105 intensity units), and C (<80×105 intensity units), respectively. (D) IFN-γ levels in pooled serum samples were measured by an ELISA. ND, not detectable. (TIF) [file pntd.0003191.s001.tif]

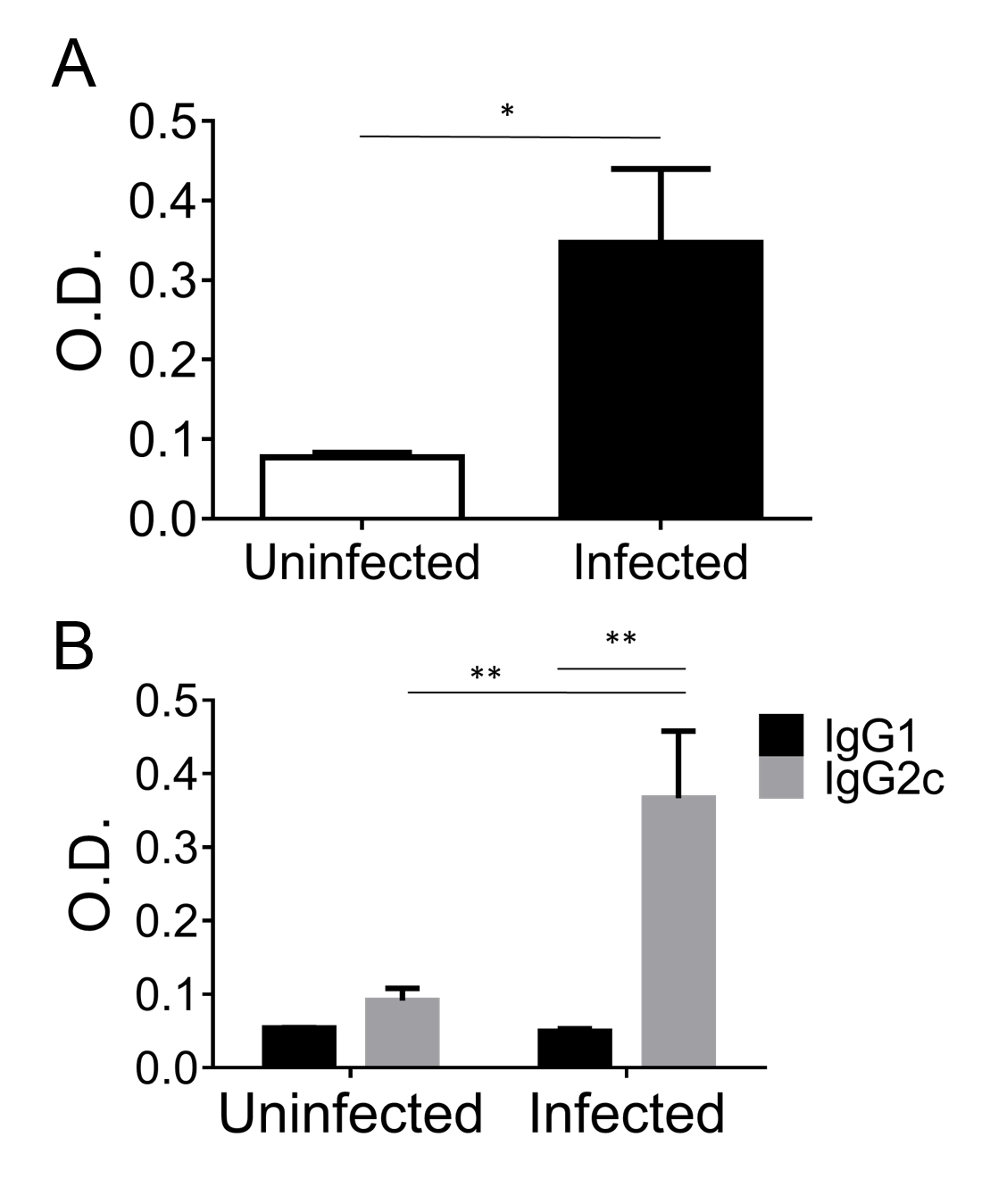

Supplement: Figure S2 — Antibody isotypes during Orientia infection. Mice (4–5/group) were inoculated with bacteria, as described in Fig. 1 . Sera were collected at 10 dpi, and antibody isotypes were determined by ELISA (IgM (A); IgG1-vs-IgG2c (B)). (TIF) [file pntd.0003191.s002.tif]

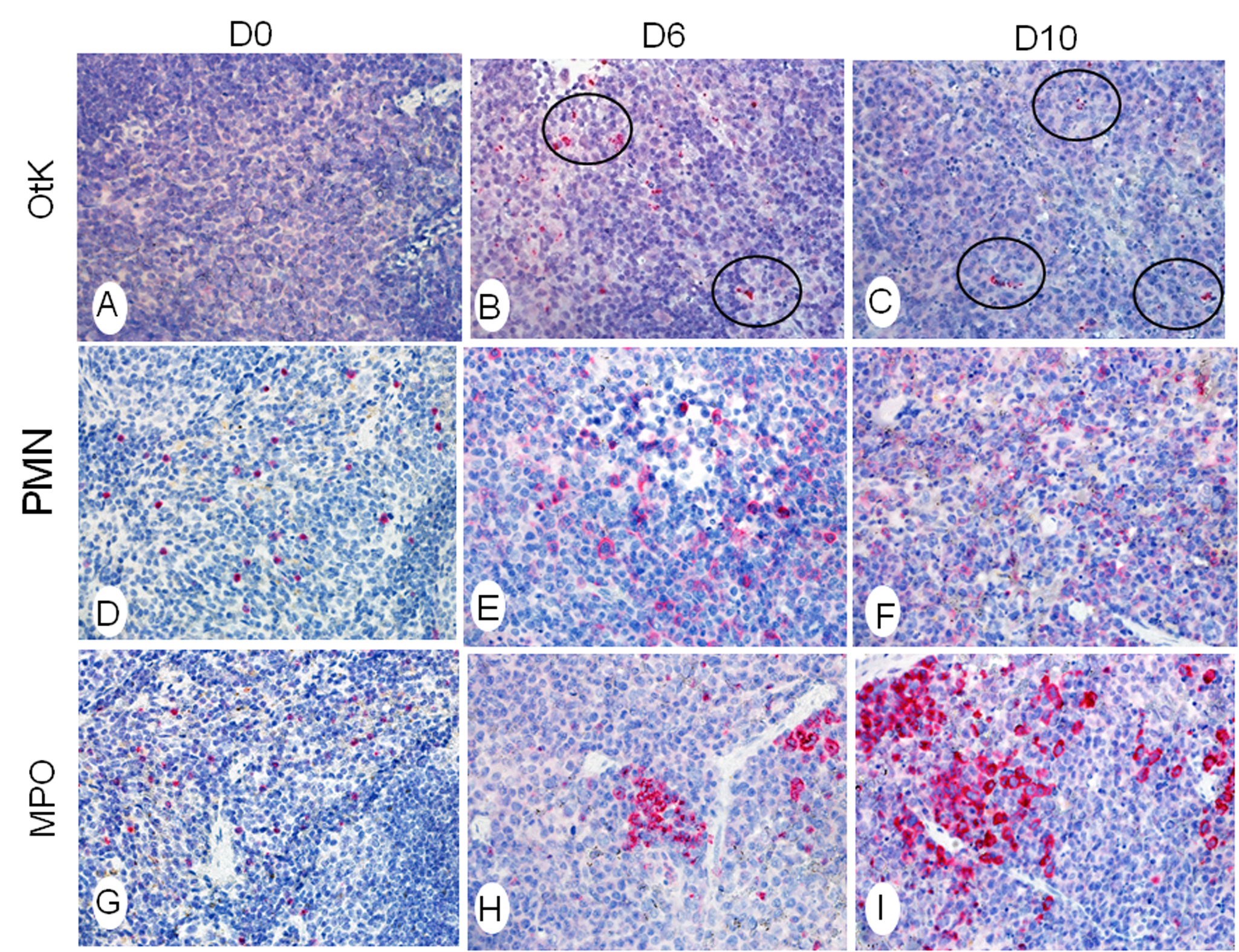

Supplement: Figure S3 — Neutrophil expansion and activation in the spleen. Mice (4–5/group) were inoculated with O. tsutsugamushi Karp stain (OtK) (A–C), as described in Fig. 1 . Spleen sections were collected at 0, 2, 6, and 10 dpi, processed, and stained by IHC for bacteria, neutrophils (PMN) (D–F), or myeloperoxidase (MPO) (G–I). Images were photographed at 40×. Positive staining is in red. Examples of bacterial staining are marked in circles. (TIF) [file pntd.0003191.s003.tif]

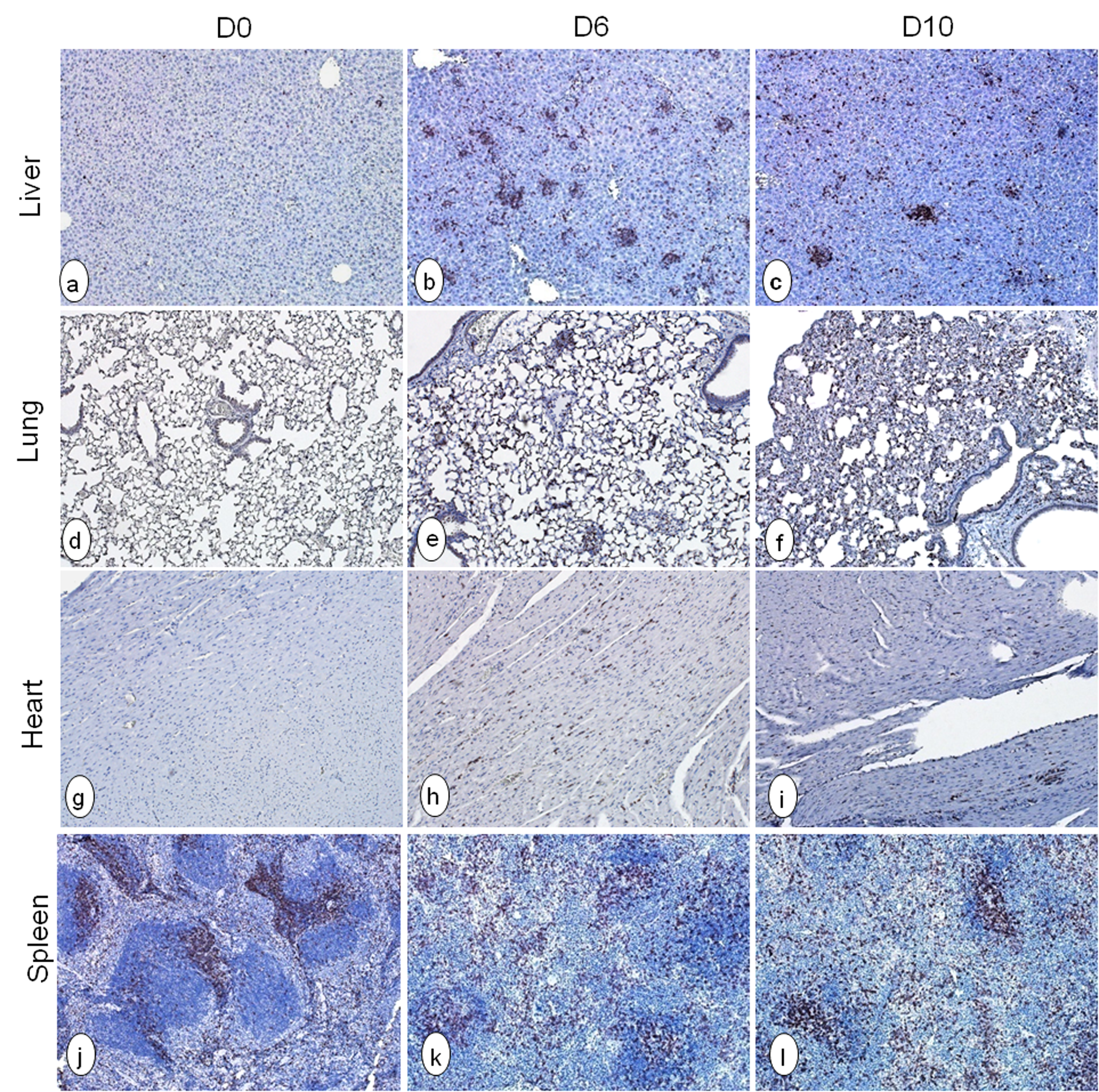

Supplement: Figure S4 — CD3+ T cell distribution in O. tsutsugamushi -infected tissues. Mice (4–5/group) were inoculated with O. tsutsugamushi, as described in Fig. 1 . Tissue sections were collected from the liver (a–c), lungs (d–f), heart (g–i), and spleen (j–l) at 0, 6 and 10 days post-infection and stained for anti-CD3. Images were photographed at 10×. The most remarkable changes were: 1) the extensive distribution of CD3+ T cells in the liver at 10 dpi (in comparison to 6 dpi), 2) the marked loss of alveolar space in the lungs at 10 dpi (due to extensive inflammatory responses), and 3) the extensive reorganization of T- and B-cell zones in the spleen. (TIF) [file pntd.0003191.s004.tif]
