# Supplementary material for: Strong Type 1, but Impaired Type 2, Immune Responses Contribute to Orientia tsutsugamushi-Induced Pathology in Mice
Source: PLoS Negl Trop Dis. 2014 Sep 25;8(9):e3191. doi: 10.1371/journal.pntd.0003191 (PMC4177881; doi:10.1371/journal.pntd.0003191)
Supplement: Table S1 — Real-time PCR primers of murine genes. The primer sequences for mouse genes analyzed in this study are listed (5′ to 3′ direction). (DOCX) [file pntd.0003191.s005.docx]

| **Supplemental Materials** | | | |
| --- | --- | --- | --- |
|  |  |  |  |

**Table. S1 Real-time PCR primers of murine genes**

Forward (5’ to 3’) Reverse (5’ to 3’)

ANG1 ATCCCGACTTGAAATACAACTGC CTGGATGATGAATGTCTGACGAG

ANG2 GGTTGCTATCCGTAAAGAAGAGC GGGGAAGGTCAGTGTGTAGATG

CXCL9 GGAGTTCGAGGAACCCTAGTG GGGATTTGTAGTGGATCGTGC

CXCL10 CCAAGTGCTGCCGTCATTTTC GGCTCGCAGGGATGATTTCAA

IFN-γ ATGAACGCTACACACTGCATC CCATCCTTTTGCCAGTTCCTC

IL-4 GGTCTCAACCCCCAGCTAGT GCCGATGATCTCTCTCAAGTGAT

IL-6 TAGTCCTTCCTACCCCAATTTCC TTGGTCCTTAGCCACTCCTTC

IL-10 GCTCTTACTGACTGGCATGAG CGCAGCTCTAGGAGCATGTG

IL-13 CCTGGCTCTTGCTTGCCTT GGTCTTGTGTGATGTTGCTCA

IL-33 TCCAACTCCAAGATTTCCCCG CATGCAGTAGACATGGCAGAA

ST2 TGTATTTGACAGTTACGGAGGGC ACTTCAGACGATCTCTTGAGACA

TNF-α ATAGCTCCCAGAAAAGCAAGC CACCCCGAAGTTCAGTAGACA

GAPDH TGGAAAGCTGTGGCGTGAT TGCTTCACCACCTTCTTGAT

β-actin CGAGGCCCAGAGCAAGAGAG CGGTTGGCCTTAGGGTTCAG

**Table S1. Real-time PCR primers of murine genes.** The primer sequences for mouse genes analyzed in this study are listed (5’ to 3’ direction).
